# Microrobots for Antibiotic-Resistant Skin Colony Eradication

**DOI:** 10.1021/acsami.5c08683

**Published:** 2025-06-25

**Authors:** Anna Jancik-Prochazkova, Hana Michalkova, Kristyna Cihalova, Zbynek Heger, Martin Pumera

**Affiliations:** † Future Energy and Innovation Laboratory, Central European Institute of Technology, 48274 Brno University of Technology, Purkynova 123, 61200 Brno, Czech Republic; ‡ Department of Chemistry and Biochemistry, 48269 Mendel University in Brno, Zemedelska 1, CZ 61300 Brno, Czech Republic; § Advanced Nanorobots & Multiscale Robotics Laboratory, Faculty of Electrical Engineering and Computer Science, VSB—Technical University of Ostrava, 17. listopadu 2172/15, 70800 Ostrava, Czech Republic; ∥ Department of Chemical and Biomolecular Engineering, Yonsei University, 50 Yonsei-ro, Seodaemun-gu, 03722 Seoul, Korea; ⊥ Department of Medical Research, China Medical University Hospital, China Medical University, No. 91 Hsueh-Shih Road, 40402 Taichung, Taiwan

**Keywords:** microrobots, Janus particles, biofilm, titanium dioxide, skin infection

## Abstract

Self-propelled nano- and micromachines have immense potential as autonomous seek-and-act devices in biomedical applications. In this study, we present microrobots constructed with inherently biocompatible materials and propulsion systems tailored to skin-related applications. Addressing the significant treatment challenge posed by methicillin-resistant (MRSA) skin infections, we demonstrate that photocatalytic titanium dioxide microrobots decorated with silver or platinum can effectively and rapidly eradicate MRSA biofilms grown on skin-mimicking membranes and porcine skin tissues. These microrobots are powered by hydrogen peroxide or ultraviolet light—agents considered toxic in high concentrations but commonly used in controlled amounts for skin disinfection and naturally encountered by the skin. By examining the effects of different metal coatings on the propulsion abilities of the microrobots, we show that these chemically propelled devices can eliminate biofilms without causing significant damage to the surrounding skin tissues, as confirmed by histological analysis. This work paves the way for the use of microrobots in skin-related biomedical applications, particularly in cases where traditional antibiotics are ineffective.

## Graphical abstract

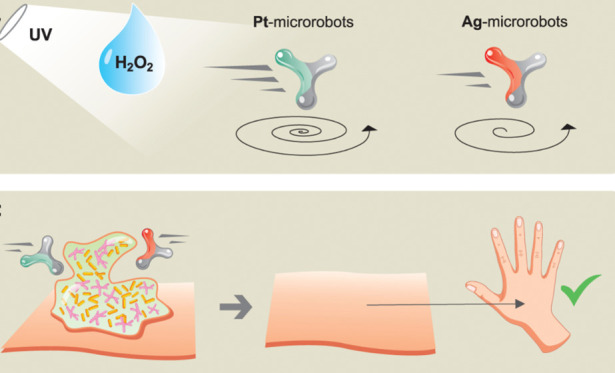

## Introduction

Tiny powerful micro-/nanorobots are gaining a great deal of attention in the field of medical applications.^1,2^ These autonomous devices can be precisely programmed to perform a specific task in a desired bioenvironment; they can be applied to visualize in imaging techniques, as sensors, to transport cargo, or to manipulate microorganisms and objects.^3^ Depending on the requirements, micro-/nanorobots can be designed using different types of materials to achieve biocompatibility, biodegradability, and scalability alongside the desired functionality.^4^

Considering medical applications, the use of micro-/nanorobots has been shown to be very convenient for bacterial infections and biofilm treatment.^5,6^ Bacterial biofilms represent a serious health risk; they can grow on natural (teeth, mucosa, skin, and organs) and artificial surfaces (implants, catheters, and devices), causing infections that are extremely challenging to treat, especially in the case of biofilms formed by antibiotic-resistant strains.^7^ The biofilm typically consists of not only microorganisms but also extracellular polymeric substances that act as a protective matrix, making biofilm eradication difficult. In most cases, the biofilm must be removed mechanically in conjunction with antibiotic treatment.^8,9^ In case of the infection of tissues of living organisms, the strategy of applying antibacterial wound healing covers seems to be efficient.^10–12^ However, in this case, the application has a preventive character to eliminate bacteria and the further spread of infection.^13–15^ On the other hand, the advantage of using micro-/nanorobots in biofilm treatment is their dynamic action; micro-/nanorobots can be actively navigated to access confined spaces, such as catheters, root canals, titanium implants, etc., and mechanically eradicate the already grown biofilm.^16,17^ Recently, the combination of catalytic abilities with mechanical eradication has been shown to be a highly efficient approach to biofilm treatment in confined spaces.^18,19^ Moreover, the nano-/microrobots can act as drug delivery systems, enabling both mechanical biofilm disruption as well as antibiotics local treatment.^20^

The use of microrobots for medical applications is still considered controversial, particularly in terms of safety. One of the burning issues is the search for an efficient and biocompatible universal propulsion mechanism. Propulsion is typically induced (i) chemically by using chemical fuels to generate chemical and electrochemical gradients as the driving force of propulsion abilities or (ii) by an external field, such as light irradiation, sound, magnetic or electric field, among others.^21–24^ From this point of view, it is important to note that a universal approach to designing micro-/nanorobots for biorelated and medical applications does not exist. Each application needs to be considered separately and, eventually, the compromise between biocompatibility, propulsion, and cost efficiency must be evaluated.^25^ For example, micro-/nanorobots driven by urea can be used conveniently in the bladder environment where urea, acting as a fuel, naturally occurs.^26^ Magnetically driven microrobots can be used in deeper tissues by applying external magnetic systems.^27^ Even hydrogen peroxide (H_2_O_2_)-driven micro-/nanorobots, which are especially controversial for their use in biorelated applications due to the toxic nature of H_2_O_2_, have shown great potential in biofilm treatment.^28^ Their applications in the oral cavity have been broadly explored as H_2_O_2_ is typically applied as a commercially available wound disinfectant up to a concentration of 3 wt %.^29^ By tailoring the design of the microrobots specifically for the desired application, the concentration of H_2_O_2_ can be eventually reduced by choosing photocatalytic materials, with lower toxicity to the bioenvironment.^30^ In addition to dental applications, the use of microrobots for the treatment of skin bacterial infections has shown great potential, particularly to treat skin ulcers^31^ and abscesses.^32^

Here, we fabricated TiO_2_-based Janus-type microrobots partially decorated with platinum or silver (referred to as Pt-microrobots and Ag-microrobots, respectively) to evaluate their use for bacterial skin infections (Scheme 1). Taking advantage of the fact that skin-related infections are commonly treated with H_2_O_2_, we used this chemical as a fuel to propel the microrobots to study their ability to eradicate bacterial biofilms. Exploiting this synergy between H_2_O_2_ and the bioactivity of microrobots, we also explored the influence of the chosen metal. Whereas platinum is more typical as the catalytic portion in the architectonics of microrobots, silver is commonly applied in biomedical applications for its antimicrobial properties. The obtained data revealed that both types of microrobots showed efficiency in biofilm eradication using traditional microplates as well as a skin-mimicking membrane.

**Scheme 1:**
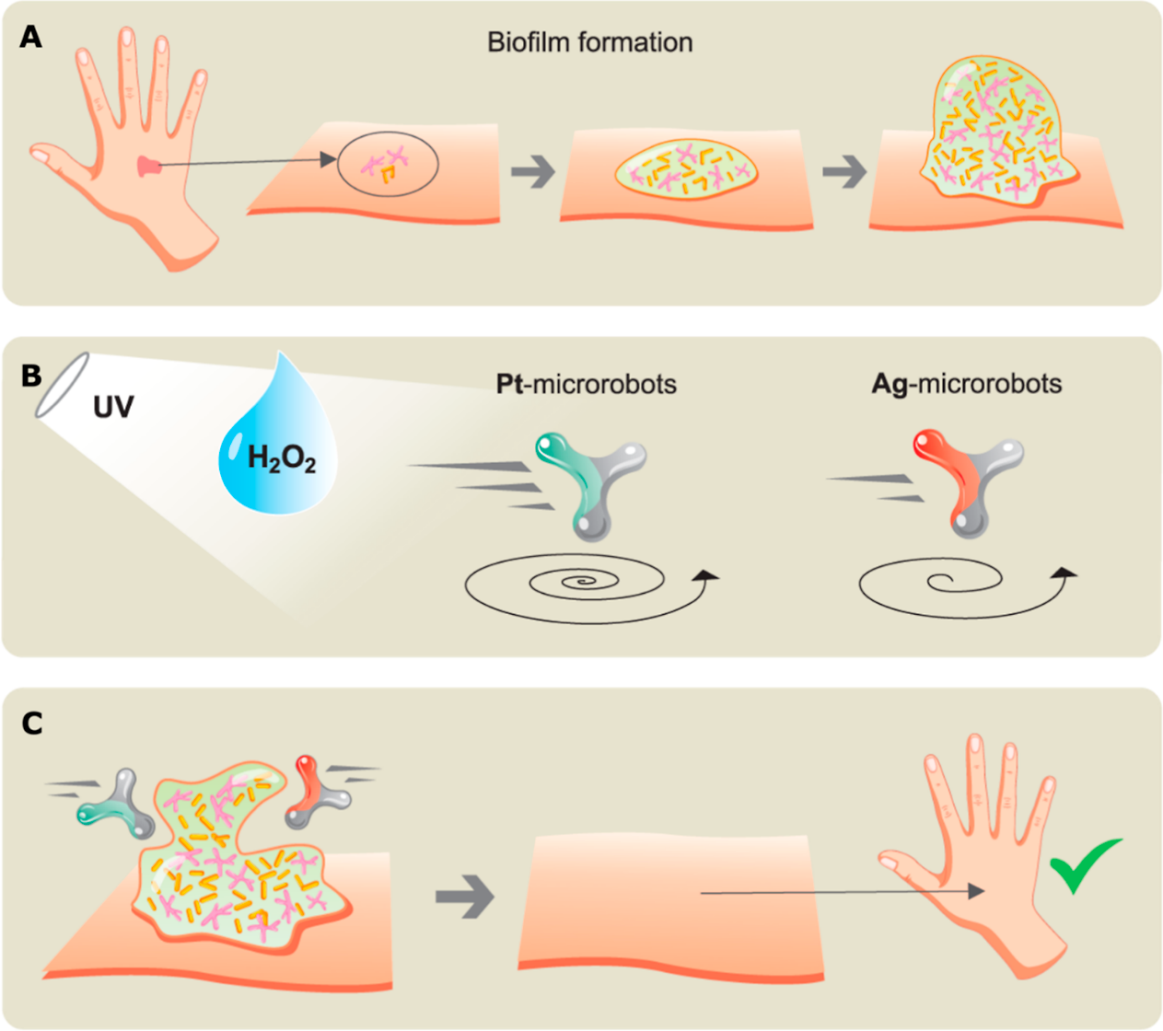
A Schematic Illustration of Biofilm Formation and Removal^a^

a (A) Biofilm growth on an infected skin wound. (B) Propulsion abilities of Pt- and Ag-microrobots under UV irradiation in the presence of H_2_O_2_ as a fuel. (C) The use of Pt- and Ag-microrobots for biofilm eradication on the skin model.

## Results and Discussion

The microrobots were prepared from commercially available rutile titanium oxide (TiO_2_) microparticles of irregular morphology (<5 μm). The crystalline phase of the TiO_2_ microparticles was verified by recording a powder X-ray diffractogram (Figure S1). The diffractogram revealed the presence of additional peaks at 25.3° and 48.0° that suggested a minor proportion of the coexistence of an anatase phase within the material. As the anatase phase is commonly used in the field of nano-/microrobotics for its photocatalytic properties that contributes to the propulsion abilities,^33^ we used the material without any further modifications. To tailor the microrobots specifically for skin biofilm eradication in the next step, the surface of the rutile microparticles was nonhomogeneously coated with a platinum or silver layer of 30 nm to form Janus-like microrobots; the resulting microrobots are referred to as Pt-microrobots and Ag-microrobots, respectively. The resulting morphology is presented in Figure S2; the individual microrobots were irregular aggregates of rutile microparticles.

To characterize the morphology of microrobots in detail, scanning electron microscopy (SEM) was performed, along with energy-dispersive X-ray (EDX) spectroscopy characterization. Figures 1A and 1B show typical morphologies of a single Pt-microrobot and Ag-microrobot, respectively, and their elemental composition, which proves a successful partial decoration with platinum and silver, respectively. Due to the irregular character of individual microrobots, no regular coating was achieved. Instead, areas of metallic coating are irregularly present within the structure of microrobots, thus not forming traditional Janus microrobots with a clearly defined body and a metallic cap. However, for the purposes of the application in the field of microrobotics, the coating is supposed to bring asymmetry to the structure of microrobots to enhance the propulsion abilities, which was accomplished as discussed further. The corresponding spectra showing comprehensive results of the elemental mappings are presented in Figure S3.

**Figure 1:**
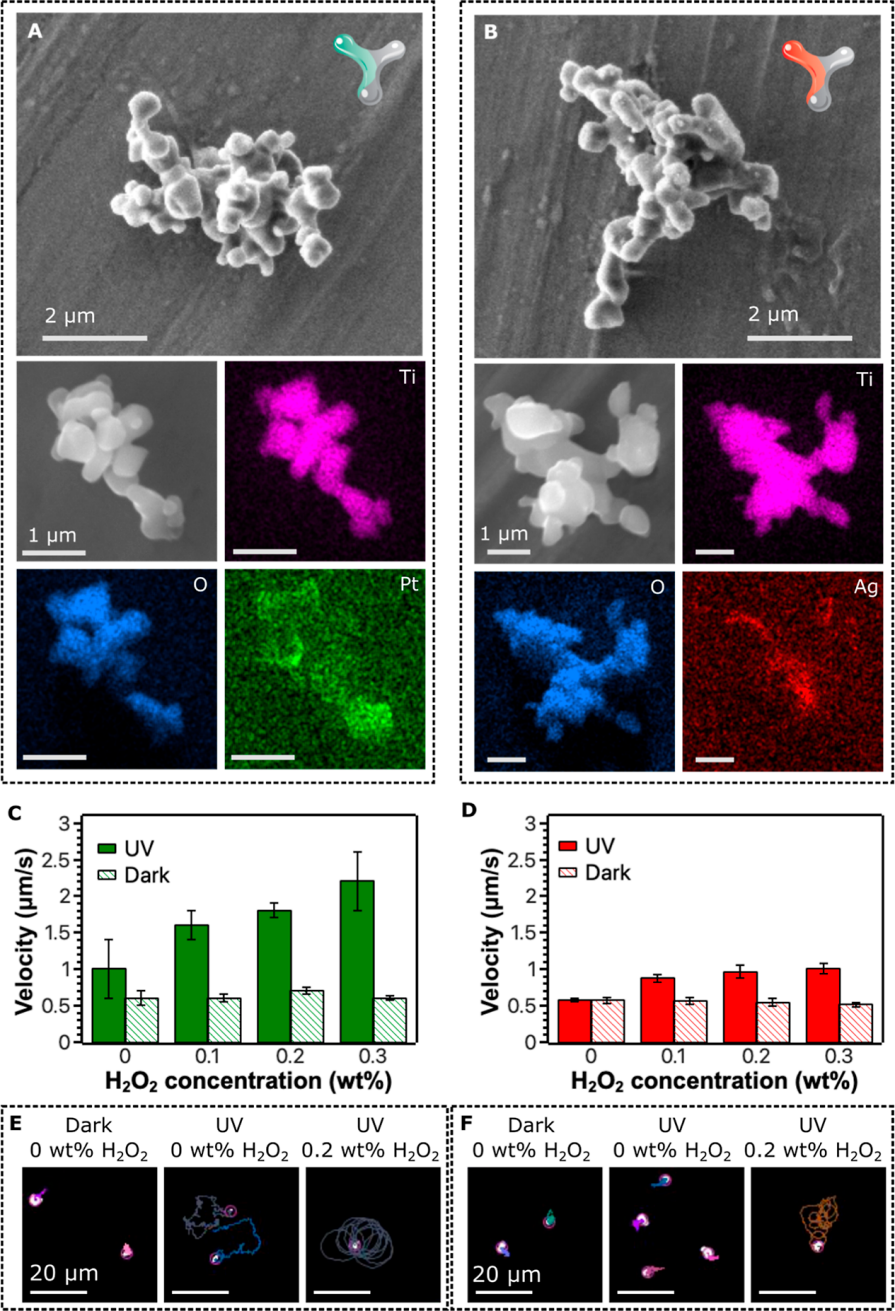
Structural characterization of microrobots. SEM micrographs of (A) Pt-microrobots and (B) Ag-microrobots. EDX mapping is included to demonstrate the elemental composition. Average velocities of (C) Pt-microrobots and (D) Ag-microrobots as a function of H_2_O_2_ concentration and UV light irradiation. (E,F) Typical trajectories of Pt-microrobots and Ag-microrobots, respectively, as a function of the concentration of H_2_O_2_ as the fuel and light irradiation (trajectories were monitored for 10 s).

The propulsion of Janus-type microrobots is typically induced using chemical fuels such as H_2_O_2_ that catalytically decomposes over the metallic layer. Fuel decomposition leads to the generation of a nonhomogeneous chemical gradient, which is the driving force for the propulsion of the microrobots. Taking into account the chemically driven propulsion in our case, the microrobots were first fueled with 3 wt % H_2_O_2_. The concentration of 3 wt % was chosen because it is commercially available as a disinfectant for the treatment of surface skin wounds. As expected, Pt-microrobots were propelled in the presence of 3 wt % H_2_O_2_ (Figure S4). This observation suggests that H_2_O_2_ is successfully catalytically decomposed on the Pt surface, leading to the formation of a local oxygen gradient that is a driving force for propulsion due to self-diffusiophoresis.^34^ On the contrary, silver did not induce the propulsion of Ag-microrobots in this setting.

TiO_2_-based microrobots were previously reported to be propelled under light irradiation due to their photocatalytic properties whereby they can decompose H_2_O_2_ fuel, typically under ultraviolet (UV) irradiation, which induces propulsion abilities. In more detail, propulsion abilities come from photocatalytic decomposition of H_2_O_2_ over generated holes in the TiO_2_ material, forming proton and oxygen gradients. In this specific case, the mechanism of the propulsion abilities lies in self-electrophoresis. Naturally, the catalytic effect of platinum and the photocatalytic abilities of TiO_2_ can work in synergy, causing an acceleration of TiO_2_/Pt Janus microrobots under UV irradiation.^35^ Bearing this in mind, we assumed that the synergy would allow us to apply a lower concentration of H_2_O_2_ compared to purely nonelectrochemical decomposition of fuel-driven propulsion. The reason for reducing the fuel concentration is that the use of H_2_O_2_ is considered harmful to cells, especially at a concentration of 3 wt %, as originally tested in this study.^36^ Figures 1C and 1D show the propulsion abilities of Pt-microrobots and Ag-microrobots, respectively, as a function of UV light irradiation and H_2_O_2_ concentration. Clearly, 0.3 wt % H_2_O_2_ was not enough to propel the microrobots under dark conditions. However, the propulsion abilities were significant under UV light irradiation. In the case of Pt-microrobots, propulsion was apparent only under UV irradiation, leading to an average velocity of 1.0 ± 0.4 μm/s. Applying H_2_O_2_ as the fuel even enhanced the propulsion abilities, where the velocity increased up to 2.2 ± 0.4 μm/s in the presence of 0.3 wt % of H_2_O_2_. The propulsion of Ag-microrobots was not observed when only UV irradiation was applied without any fuel; the microrobots were successfully propelled after fuel addition. However, the average velocity was lower compared to the Pt-microrobots; the average velocity was 1.0 ± 0.1 μm/s in the presence of 0.3 wt % of H_2_O_2_. Typical recorded trajectories of the microrobots are demonstrated in Figure 1E,F for Pt-microrobots and Ag-microrobots, respectively. Mean squared displacement (MSD) curves of representative trajectories are presented in Figure S5.

Encouraged by the promising propulsion capabilities of both types of microrobots, along with their bactericidal effects and antibiofilm activity, they were initially tested using well plates (Figure 2A) to evaluate whether the influence of the metal choice and the efficiency of motion can be reflected in the antibiofilm activity. For this purpose, a strain of methicillin-resistant (MRSA) was cultured as a model biofilm system. Biofilm eradication was tested in propelled modes (referred to as “propelled mode”) by applying different concentrations of microrobots in the presence of 0.2 wt % H_2_O_2_ in addition to UV irradiation for 30 min. As a control experiment, the biofilm was exposed to microrobots in static mode, i.e., without applying fuel or UV irradiation. As demonstrated in Figure 2, biofilm eradication was efficient using both types of microrobots. However, some differences were observed when the efficiency of Pt- and Ag-microrobots was compared. When Pt-microrobots were applied in static mode in the concentration range 10–200 μg/mL, the viability exceeded 100%, which suggests that biofilm growth was supported by the presence of microrobots (Figure 2B). In contrast, the presence of static Ag-microrobots did not support the biofilm growth to such a great extent in the same experimental setting. The concentration range of 50–200 μg/mL of Ag-microrobots did not significantly influence bacterial viability. The concentration of 10 μg/mL of Ag-microrobots caused a slight increase in biofilm viability. The stimulation of biofilm growth by static Pt- and, to a lesser extent, Ag-microrobots can plausibly be attributed to hormesis—an adaptive response in organisms triggered by low-level stress that enhances their overall fitness.^37^ However, when the fuel and UV irradiation were applied to activate the propelled mode, effective biofilm eradication was achieved, with Ag-microrobots showing a higher efficiency in bacterial biofilm eradication. When applying the concentration of Ag-microrobots of 100 μg/mL in the propelled mode, the viability was 14 ± 1% (Figure 2C). In comparison, the viability of the biofilm when Pt-microrobots were used at the same concentration was 98 ± 11% (Figure 2B). Biofilm eradication was further monitored with confocal microscopy (Figure 2D); in this case, the microrobots were applied in propelled and static modes at a concentration of 100 μg/mL. Similar to the observation already discussed, the efficiency of Ag-microrobots in biofilm eradication was more significant. This observation is not surprising, considering the antibacterial effect of silver. Recently, Ussia et al.^19^ demonstrated the efficiency of Ag/TiO_2_ Janus nanorobots in biofilm eradication in dental implants. The efficiency in biofilm eradication was explained by the fact that silver tends to passivate with Ag^+^ ions, which possess antimicrobial effects. Clearly, the antibacterial effect does not originate solely from the effect of silver. Previous studies^20,38^ have suggested the crucial contribution of the mechanical eradication originating from the propulsion of the microrobots. In addition, the use of photocatalytic TiO_2_ as the core material allows generation of reactive oxygen species under UV irradiation that contribute to a significant antibacterial activity.^39,40^

**Figure 2:**
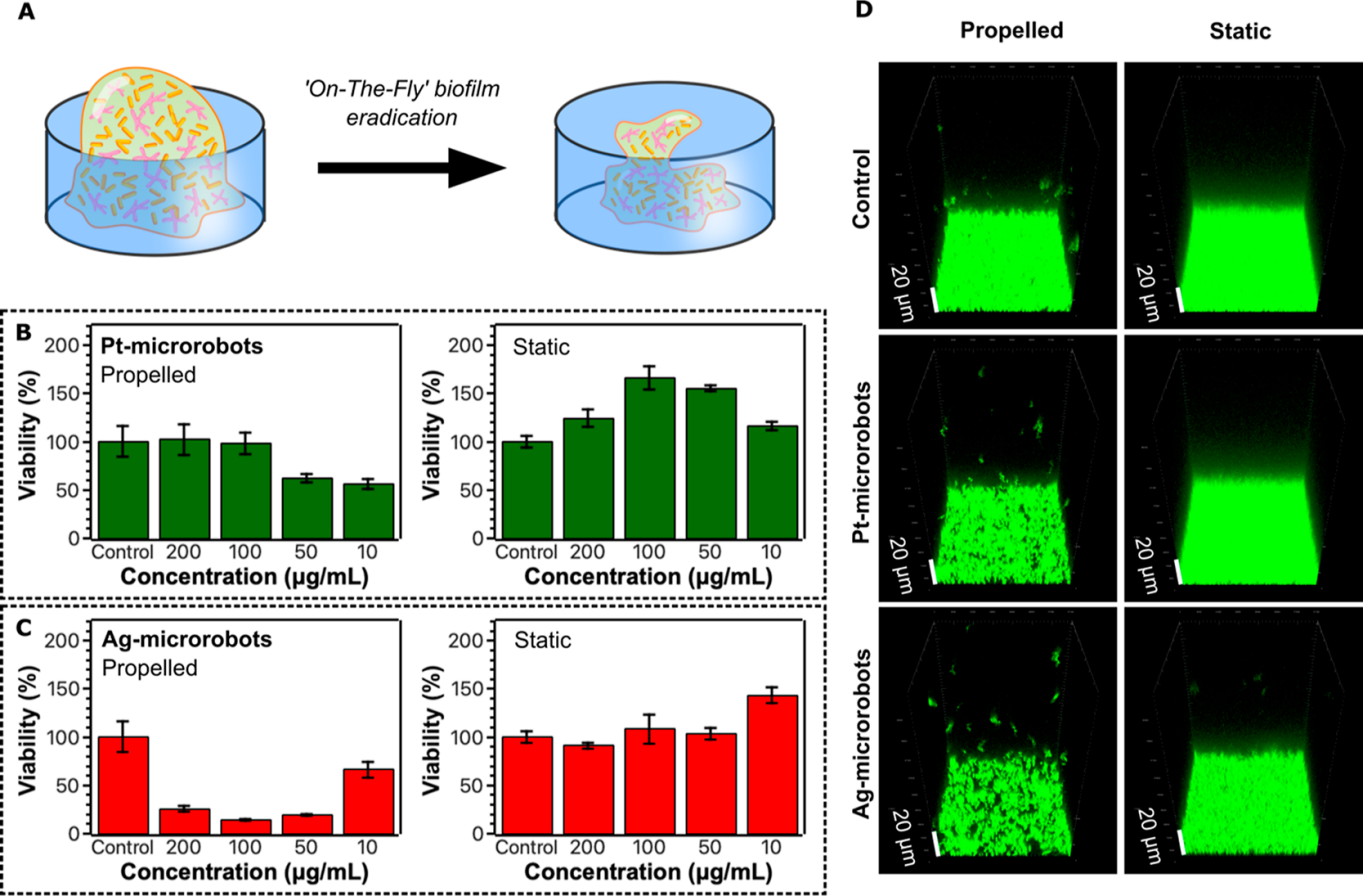
Eradication of biofilms using Pt-/Ag-microrobots. (A) Illustration of “on-the-fly” eradication of biofilms. Viability of biofilms using (B) Pt-microrobots and (C) Ag-microrobots, and comparison of propelled and static conditions after 30 min of exposure. Propelled conditions were achieved by the addition of 0.2 wt % H_2_O_2_ as the fuel alongside UV irradiation. (D) Confocal fluorescence micrographs showing the degree of biofilm eradication after treatment with microrobots in propelled and static modes for 30 min. The concentration of microrobots was 100 μg/mL, and the propelled mode was achieved by applying 0.2 wt % H_2_O_2_ and UV irradiation.

It should be noted that exposing the biofilms to propelled Ag-microrobots of the concentration of 100 μg/mL for 15 min caused the viability to drop to 32 ± 2% (Figure S6). Interestingly, in the case of Pt-microrobots, the viability was 205 ± 5% after 15 min, indicating that biofilm eradication was not efficient. On the contrary, these results could suggest potential support in biofilm growth by Pt-microrobots despite the application of H_2_O_2_ and UV irradiation.

Treating bacterial biofilms on the skin also includes a possible interaction of microrobots with skin cells; therefore, the viability of the immortalized human keratinocyte cell line (HaCaT) was studied in the presence of Pt- and Ag-microrobots (Figure S7). First, cell viability was studied as a function of microrobot concentration during 72 h incubation to evaluate the potential long-term toxicity of the microrobots themselves. As a result, Ag-microrobots showed higher toxicity compared to Pt-microrobots in the long-term toxicity study without applying any fuel or UV irradiation. The IC_50_ value of Ag-microrobots was determined to be approximately 200 μg/mL after 72 h of exposure. This concentration is notably higher than values reported in the literature for similar nanomaterials,^41–43^ indicating low cytotoxicity of our microrobots toward normal epithelial cells. On the contrary, the IC_50_ value for Pt-microrobots in the concentration of 200 μg/mL was not reached; the viability of 79 ± 2% suggested better compatibility with normal human cells. In agreement with the results of the biofilm eradication experiments, we considered exposure of skin tissue to a microrobot concentration of 100 μg/mL for 30 min. Therefore, the exposure of skin cells to microrobots in both propelled and static modes was monitored for 30 min. As suggested in Figure S7B, the propelled microrobots caused significant inhibition of cell culture viability. Specifically, the viability of HaCaT cells was determined as 23 ± 1% and 20 ± 2%, respectively, when applying Pt-microrobots and Ag-microrobots in propelled mode. This effect could be attributed to the use of H_2_O_2_ as a fuel. However, it should be noted that in skin disinfection procedures, H_2_O_2_ is typically used in significantly higher concentrations of up to 3 wt %. At these concentrations, H_2_O_2_ was shown not only to have disinfection abilities but also to act as a signaling molecule that improves dermal wound healing without causing tissue damage.^44^ The HaCaT cell monoculture does not fully represent the complex microenvironment and anatomy of human skin surface cells. Thus, these data must be taken with a grain of salt and other advanced experimental models employed to fully delineate the applicability of H_2_O_2_-propelled microrobots for in vivo applications.

Encouraged by the efficiency of biofilm eradication using microrobots, we further tested biofilm eradication on a skin-mimicking poly­(ether sulfone) membrane (Figure 3A). In this case, the efficiency of biofilm removal was monitored by determining the degree of coverage of the area after treatment with microrobots using digital microscopy. As expected, biofilm eradication was more efficient in propelled mode compared to static mode (Figure 3B), corroborating previous experiments. Simultaneously, the use of Ag-microrobots was more efficient than that using Pt-microrobots. In particular, the average biofilm coverage was 25 ± 5% after applying Ag-microrobots for eradication, while the application of Pt-microrobots resulted in an eradication coverage of 59 ± 2%. This observation is also supported by the presentation of micrographs obtained by confocal microscopy. The top view on the surface of the membrane clearly shows the efficiency in biofilm removal (Figure 3C). Biofilm eradication was more efficient in the case of using Ag-microrobots, even when studying the system on the skin-mimicking membrane. It could be concluded that along with mechanical degradation of the biofilms, the antimicrobial activity of silver has a synergistic effect. In this regard, the highest possible speed might not be the crucial parameter when microrobots are applied for biofilm treatment.

**Figure 3:**
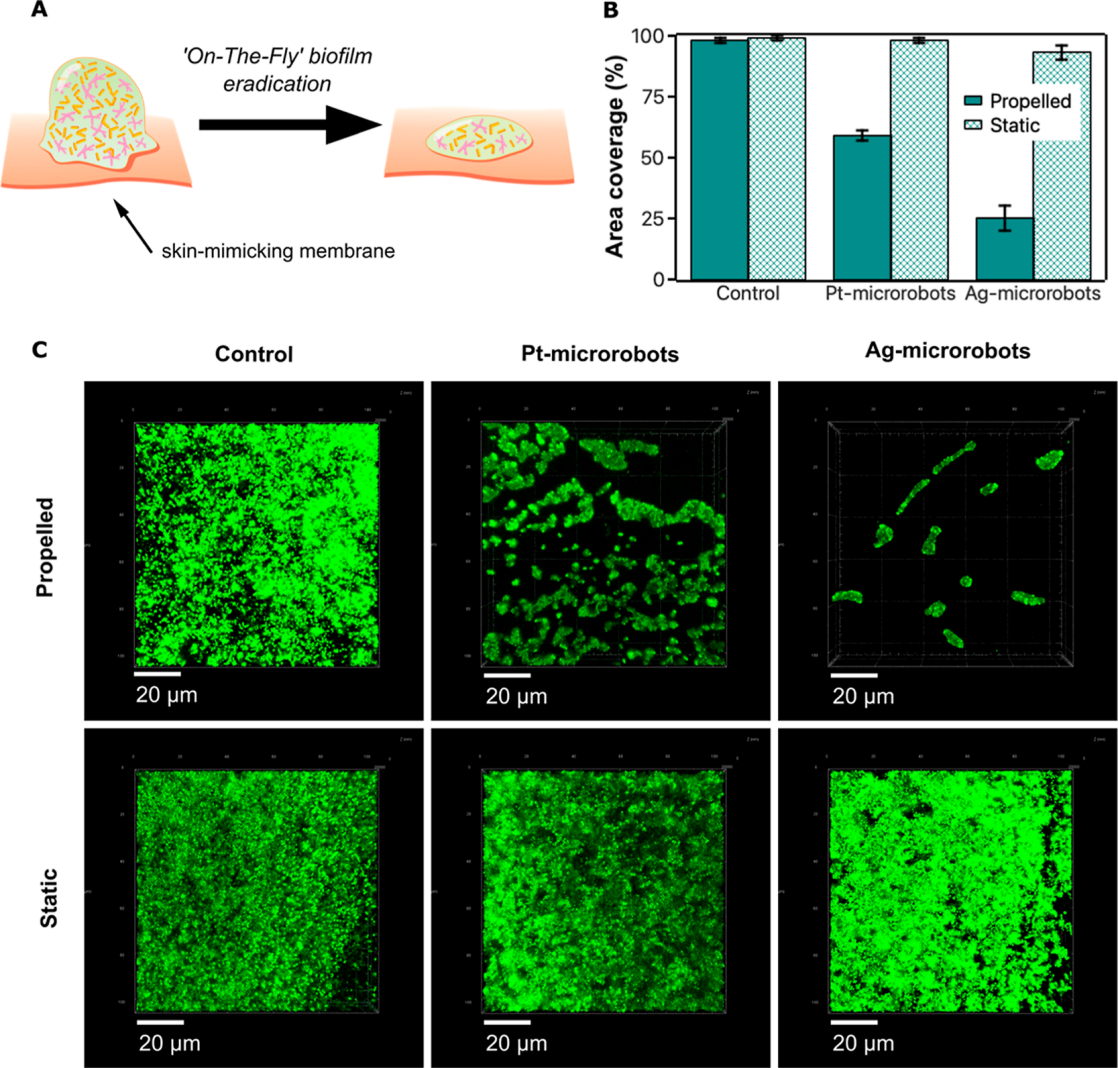
Eradication of MRSA biofilms on a skin-mimicking membrane. (A) Illustration of the eradication of biofilms on the skin-mimicking membrane. (B) Efficiency of the biofilm eradication on the skin-mimicking membrane in propelled and static modes after 30 min treatment with microrobots. (C) Fluorescence micrographs demonstrating the coverage of the biofilm on the skin-mimicking membrane after treatment with microrobots in propelled and static modes for 30 min. Micrographs show the top view on the membrane to visualize the area coverage. The propelled mode was achieved by applying 0.2 wt % H_2_O_2_ and UV irradiation.

In the next step, the possibility of eradicating biofilms from real skin tissue was tested. For this purpose, porcine skin was selected as the model (Figure 4A). The bacterial biofilm was grown on porcine skin samples ex vivo and treated with microrobots under the same conditions as in the previous experiments (0.2 wt % H_2_O_2_ along with UV irradiation to achieve biofilm eradication in the propelled mode). The samples were screened using histological procedures to visualize not only the degree of eradication of the biofilm but also the condition of the tissue. As a control experiment, the histology test of untreated porcine skin was first performed. A typical result is presented in Figure 4B. Figure 4C shows the results of the histological examination after biofilm eradication experiments. First, it should be noted that no anomalies in skin tissues were observed after biofilm eradication using microrobots, suggesting that the overall tissue was intact after treatment. The biofilm was successfully eradicated when treated with microrobots. A certain degree of biofilm eradication was observed even in static mode, i.e., without applying H_2_O_2_ and UV irradiation. However, the use of microrobots in propelled mode was shown to be more efficient in agreement with previously discussed experiments. As shown in a typical histology analysis, using microrobots in propelled mode resulted in overall biofilm elimination and even complete removal in some areas. In contrast, using only static microrobots resulted in weaker and less efficient biofilm thinning, highlighting the positive role of microrobot motion in enhancing biofilm eradication. Interestingly, when the efficiency of Pt- and Ag-microrobots was compared, there was no significant difference in the biofilm disruption. Clearly, the more efficient propulsion abilities of Pt-microrobots that lead to a stronger mechanical biofilm disruption can compete with those of Ag-microrobots that possess an antibacterial effect due to the presence of silver.

**Figure 4:**
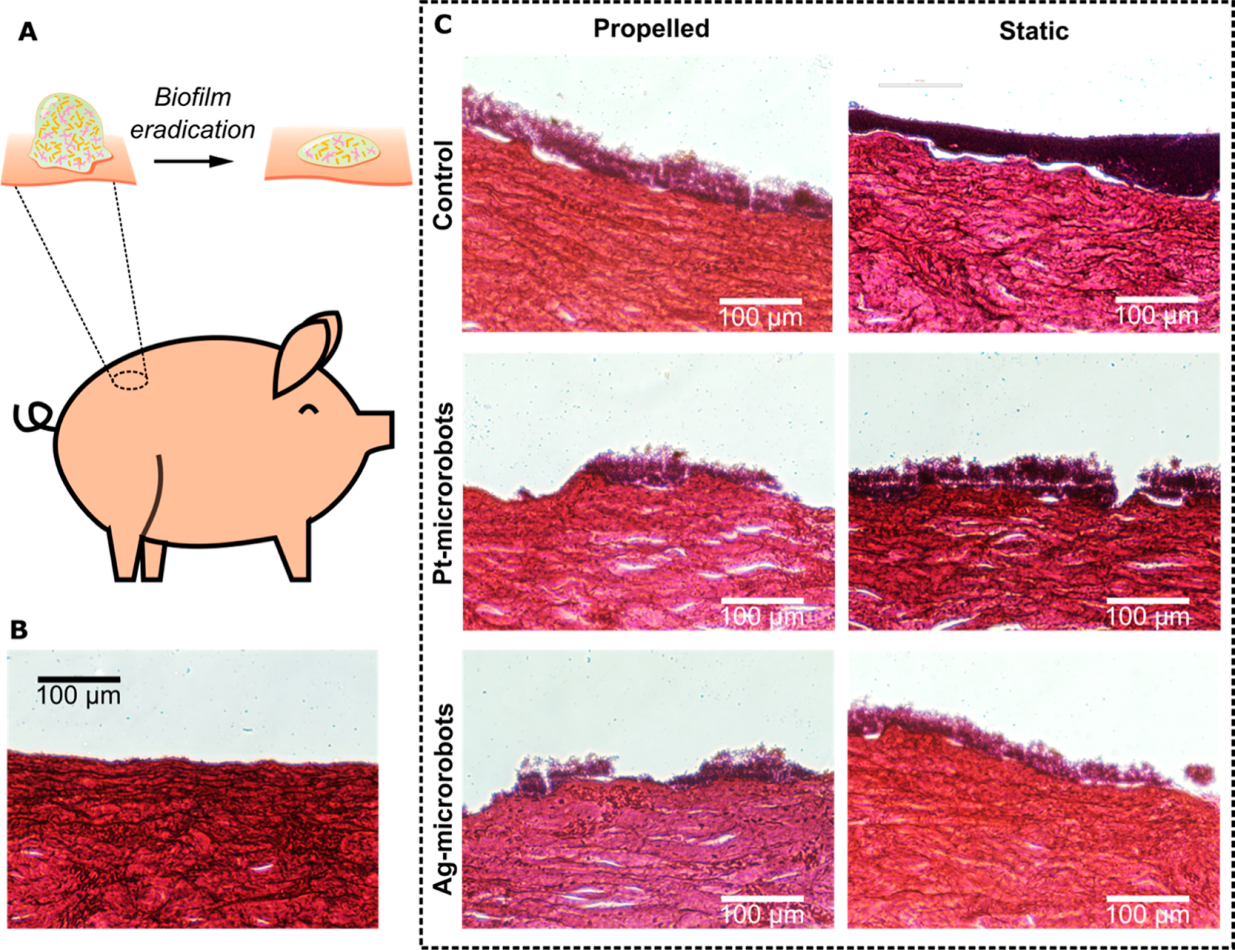
Eradication of biofilms grown on porcine skin ex vivo. (A) Illustration of the eradication of biofilms on porcine skin. (B) Control micrograph of histology showing the normal condition of porcine skin without any treatment. (C) Histological analysis of the skin treated with biofilms and the result of biofilm eradication using microrobots under propelled and static conditions after 30 min. The propelled mode was achieved by applying 0.2 wt % H_2_O_2_ and UV irradiation.

Overall results suggest the potential use of microrobots in skin tissue therapeutic applications. The “on-the-fly” mode allows biofilm disruption in areas with a limited access. It can be envisioned that the microrobots would be applied to mechanically eradicate the biofilm in confined spaces, and the subsequent application of commercial antibacterial wound dressing would provide antiseptic treatment to eliminate the skin infection and support the healing process. Such a solution is expected to provide a next-generation skin tissue therapeutic treatment.

## Conclusions

This study successfully demonstrates the use of TiO_2_-based Janus microrobots, functionalized with platinum and silver, for biofilm eradication on skin. While Pt-microrobots exhibited better propulsion, Ag-microrobots showed superior antibacterial effects, particularly in eradicating MRSA biofilms. The combination of propulsion with the antimicrobial activity of Ag-microrobots proved more efficient in biofilm removal from skin models. Histological analysis confirmed that biofilms were removed without significant damage to skin tissue, highlighting the potential for biomedical applications. The development of these microrobots offers a promising new approach for tackling antibiotic-resistant bacterial biofilms, addressing a critical challenge in modern medicine. Their ability to eradicate biofilms on skin without damaging surrounding tissues could revolutionize the treatment of infections, particularly in cases where traditional antibiotics are ineffective.

## Experimental Section

### Preparation and Characterization of Microrobots

Commercial titanium­(IV) oxide (TiO_2_, rutile) particles (<5 μm, Merck) were dispersed in ethanol (20 mg/mL) and spin-coated on glass slides. Platinum or silver was sputtered (high-vacuum coater, Leica Microsystems EM ACE 600) over the resulting TiO_2_ films in 30 nm thickness to form Janus-type microrobots. The resulting Pt- and Ag-microrobots were collected from the glass substrates by using a scalpel and kept for the experiments.

The powder XRD analysis of the starting material was performed using an X-ray powder diffractometer (Rigaku SmartLab 3 kW). Morphology of the resulting microrobots was studied by using a MIRA3 XMU (Tescan) scanning electron microscope equipped with an EDX detector (X-max, Oxford Instruments). To prepare a sample for SEM observation, microrobots were drop-cast on copper tape from their colloidal solution. The sample was coated with chromium (5 nm).

The propulsion of the microrobots was characterized using an inverted microscope (Nikon Eclipse Ti2) equipped with a digital camera (Hamatsu, C13440). UV light (361 to 389 nm, 463 mW cm^–2^) was applied using filter cubes from the white-light LED source (CoolLed, pE-300 lite). Propulsion abilities were studied on a glass slide in colloidal solution. For a typical experiment, 10 μL of a colloidal solution containing fuel in the desired concentration was placed on a glass slide and studied. Videos capturing the propulsion of microrobots were processed using ImageJ software with a TrackMate plugin to obtain typical trajectories and average speeds.

### Viability of Bacterial Biofilms

The bacterial strain used for the antibiofilm test was MRSA (CCM 7110) obtained from the Czech Collection of Microorganisms (CCM, Brno, Czech Republic). MRSA was cultured on 5% blood Columbia agar (LMS, Czech Republic) at 37 °C overnight. Fresh bacterial culture was diluted in brain heart infusion (BHI) broth with 1% glucose (Sigma-Aldrich, USA) at a density of OD_600_ = 0.1 AU. Subsequently, 100 μL of culture was placed in 10 mL of BHI, and 100 μL of this bacterial solution was grown in a U-shaped well plate at 37 °C for 7 days. The medium was refreshed every 24 h. After incubation, the grown bacterial biofilm was washed three times with phosphate-buffered saline (PBS). The ability of micromotors to disrupt the bacterial biofilm was determined in the concentration range of 10–200 μg/mL. The disruption of biofilms by microrobot movement was performed under two conditions: (i) in sterile deionized water and (ii) in sterile deionized water with 0.2 wt % H_2_O_2_ and UV irradiation (366 nm, 550 μW cm^–2^). The bacterial biofilm was exposed to the microrobots for 30 min and then rinsed three times with PBS. After the addition of Alamar Blue (Thermo Fisher Scientific, Waltham, MA, USA), the viability of the bacterial biofilm was evaluated with fluorescence measurements (560:590 nm, excitation/emission). The tests were performed in four replicates.

### Confocal Scanning Microscopy

The cultivation of the MRSA biofilm was performed as mentioned above. After incubation, 100 μL of culture was placed in 10 mL of BHI with 1% glucose, and 2 mL of this bacterial solution was grown in (i) a μ-dish (Ibidi GmbH, Planegg/Martinsried, Germany) and (ii) a membrane that mimics the human skin (poly­(ether sulfone) membrane, Strat-M, diameter 25 mm, Merck) at 37 °C for 48 h. The medium was replaced after 24 h. After incubation, the grown bacterial biofilm was washed three times with PBS and treated with the microrobots at a concentration of 100 μg/mL with 0.2 wt % H_2_O_2_ and UV irradiation (366 nm, 550 μW cm^–2^). The bacterial biofilm was exposed to microrobots for 30 min; afterward, the biofilm was rinsed three times with PBS. For the fluorescence detection of live/dead cells, a LIVE/DEAD Bacterial Viability and Counting Kit (Invitrogen) was used according to the manufacturer’s instructions. The kit contains SYTO-9 (green) for the detection of live cells and PI (red) for the detection of dead cells. Staining solution (500 μL) was added to each Ibidi μ-dish and film mimicking human skin. The samples were imaged with a Z-stack confocal laser scanning microscope (LSM 880, Carl Zeiss, Jena, Germany). SYTO9 staining was detected with an excitation wavelength of 488 nm and an emission wavelength of 490–570 nm. Propidium iodide staining was detected with an excitation wavelength of 561 nm and an emission wavelength of 566–697 nm. Stitching was performed using ZEN 3.1 software to create a single 3D *z*-stack.

### Digital Microscopy

Bacterial biofilm eradication on the surface of the miniplates was measured with a Keyence Digital Microscope VHX-5000 high-resolution zoom lens VH-Z500R/Z500T (Mechelen, Belgium). Samples were prepared as mentioned above for a film mimicking human skin, and biofilm surface coverage was determined based on the brightness threshold.

### Histology Screening

Fresh porcine skin was a kind gift from a local butcher. Skin samples were cut aseptically, disinfected using 70% ethanol, and incubated with bacterial inoculum at a density of OD_600_ = 0.1 AU (approximately 1 × 10^8^ CFU/mL) for biofilm formation as described above. After 3 days of incubation with daily medium refreshing, unattached bacterial cells were washed three times with PBS. Subsequently, the bacterial biofilm on the porcine skin surface was treated by 100 μg/mL of microrobots. Histology samples were prepared after formaldehyde fixation and paraffin embedding. Tissue sections were stained with hematoxylin and eosin and visualized with a fluorescence microscope (EVOS FL Auto 2, Olympus VS120). Exact number of remaining bacterial cells was not determined, as the applied method is not quantitative. Representative microscopic images were provided to illustrate biofilm presence or removal after the microrobots treatment.

## Supplementary Material



## Notes

XRD of starting material, SEM of microrobots, EDX spectra, tracking and MSD analysis of microrobots, and cell viability assays (PDF)
