# Supplementary material for: Microrobots for Antibiotic-Resistant Skin Colony Eradication
Source: ACS Appl Mater Interfaces. 2025 Jun 25;17(27):39340–8. doi: 10.1021/acsami.5c08683 (PMC12257449; doi:10.1021/acsami.5c08683)
Supplement: Supplementary file 1 [file am5c08683_si_001.pdf]

SUPPORTING INFORMATION

Microrobots for Antibiotic-Resistant *Staphylococcus aureus* Skin Colony Eradication

Anna Jancik-Prochazkova[1], Hana Michalkova[2], Kristyna Cihalova[2], Zbynek Heger[2], Martin Pumera[1,3,4,5*]

[1] Future Energy and Innovation Laboratory, Central European Institute of Technology, Brno University of Technology, Purkynova 123, 61200 Brno, Czech Republic

[2] Department of Chemistry and Biochemistry, Mendel University in Brno, Zemedelska 1, CZ 61300 Brno, Czech Republic

[3] Advanced Nanorobots & Multiscale Robotics Laboratory, Faculty of Electrical Engineering and Computer Science, VSB - Technical University of Ostrava, 17. listopadu 2172/15, 70800 Ostrava, Czech Republic

[4] Department of Chemical and Biomolecular Engineering, Yonsei University, 50 Yonsei-ro, Seodaemun-gu, Seoul 03722, Korea

[5] Department of Medical Research, China Medical University Hospital, China Medical University, No. 91 Hsueh-Shih Road, Taichung, Taiwan

*email: martin.pumera@ceitec.vutbr.cz

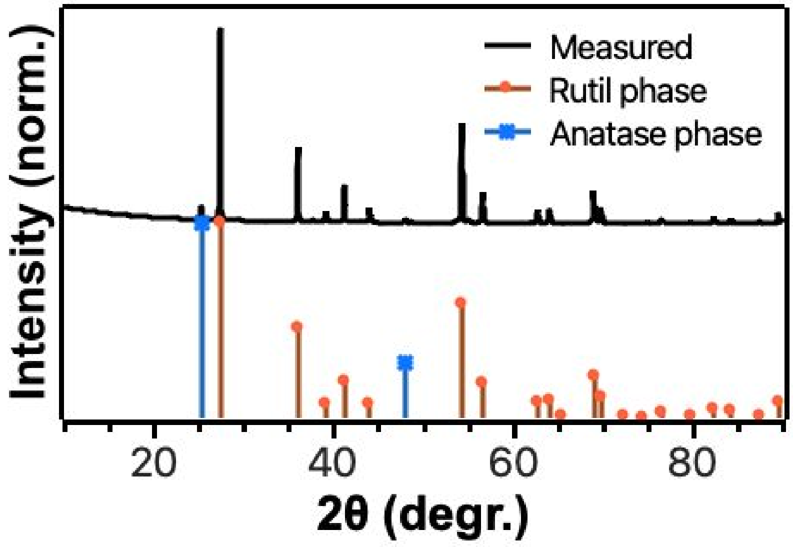

Figure S1. Powder XRD diffractogram of the starting TiO_2 material. For comparison, modelled diffractograms of rutile and anatase phase are plotted as well (PDF cards No. 9015662 and 7206075, respectively).

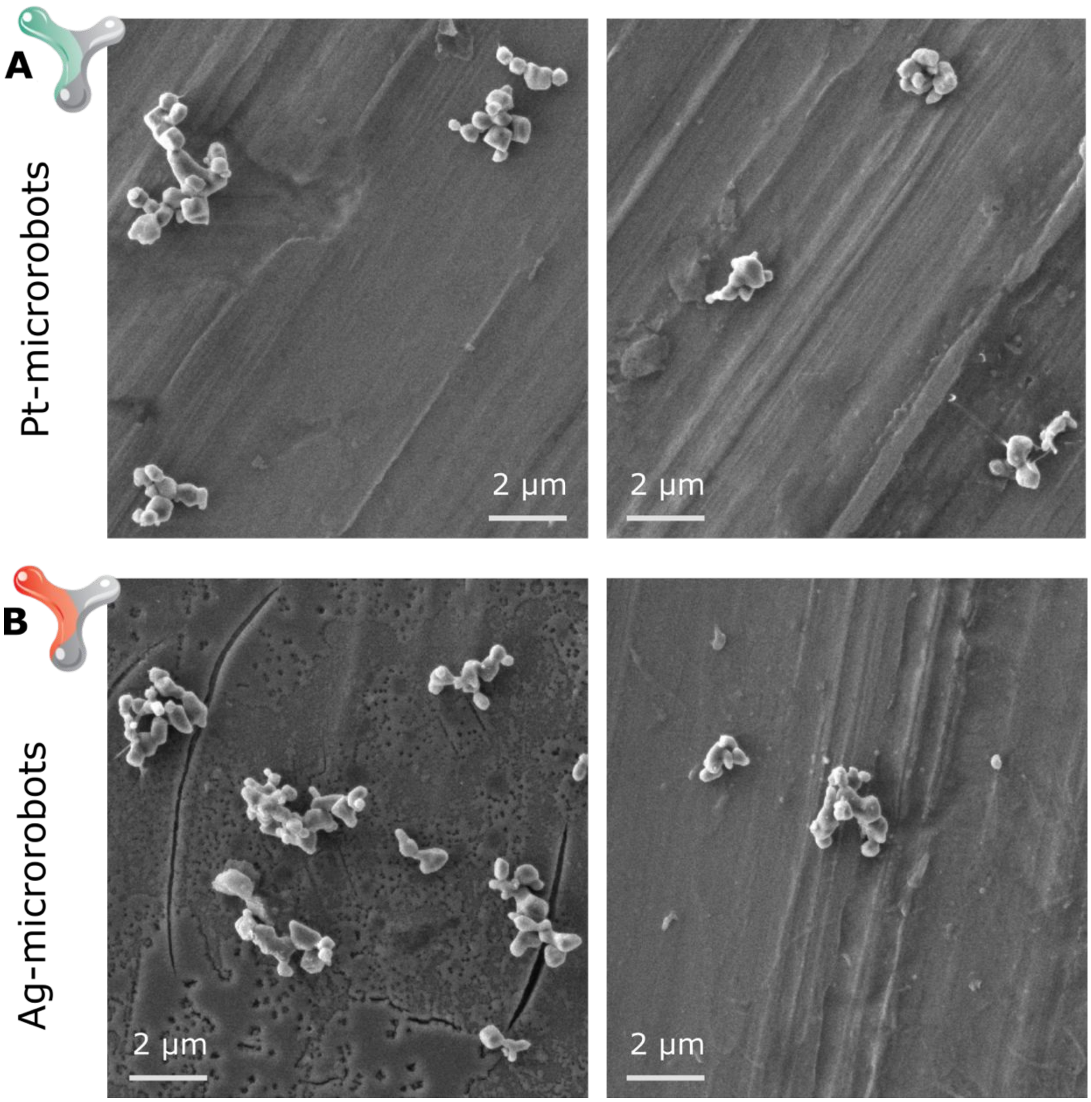

Figure S2. SEM characterization of microrobots. A, B) Micrographs of Pt-microrobots and Ag-microrobots, respectively, that demonstrate size and morphology distribution.

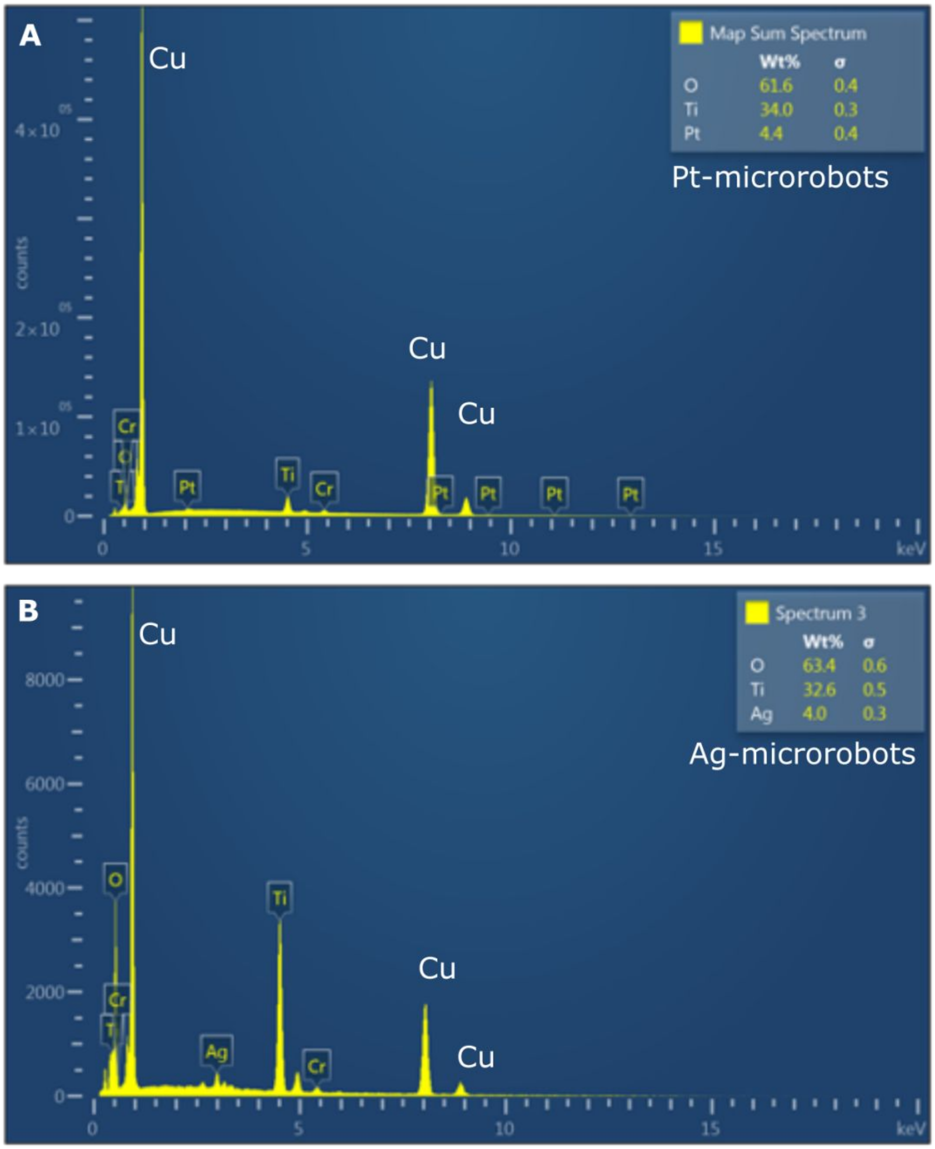

Figure S3. EDX mapping spectra demonstrating the presence of expected elements in the Pt- and Ag-microrobots (A and B, respectively). The Cu signal comes from the Cu tape that was used as a substrate for the measurement. The Cr signal comes from the sample coating with 5 nm layer of chromium.

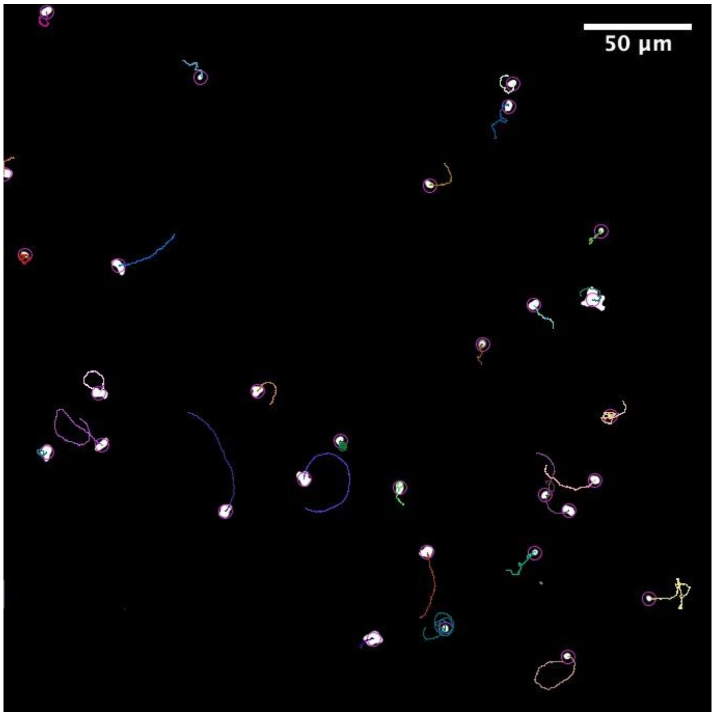

Figure S4. Tracking of Pt-microrobots fueled with 3 wt% H_2O_2 at dark conditions. The trajectories are depicted from 10 s video.

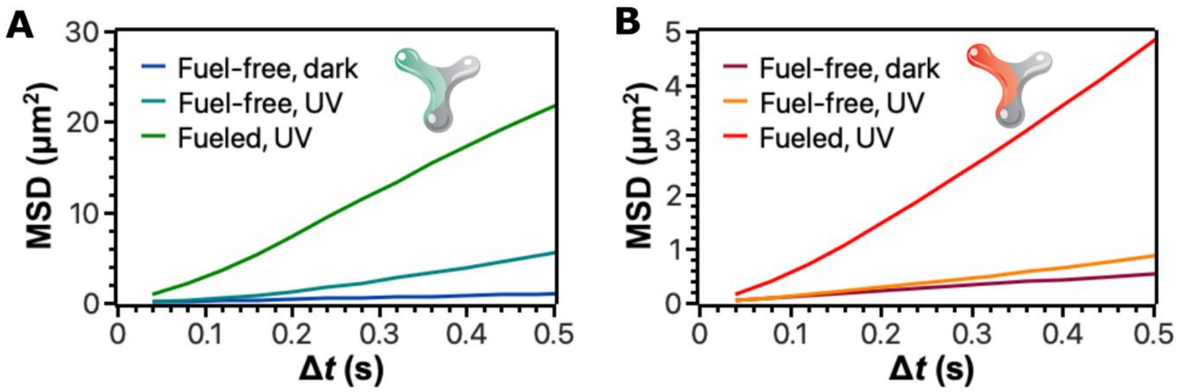

Figure S5. MSD analysis of representative trajectories of (A) Pt-microrobots and (B) Ag-microrobots at different environments. The concentration of H_2O_2 was 0.3 wt% in case of the fueled sample.

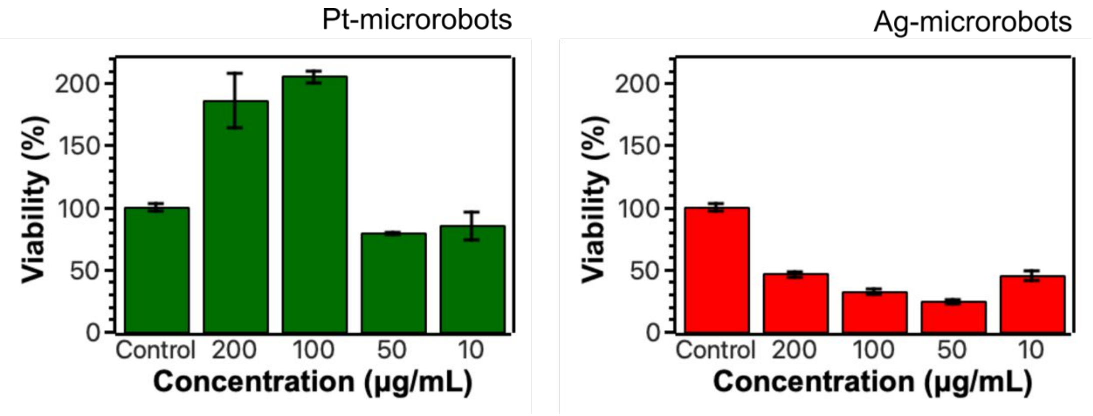

Figure S6. Viability of MRSA bacteria in biofilms treated with Pt-/Ag-microrobots of different concentrations for 15 min in the presence of 0.2 wt% H_2O_2 under UV irradiation.

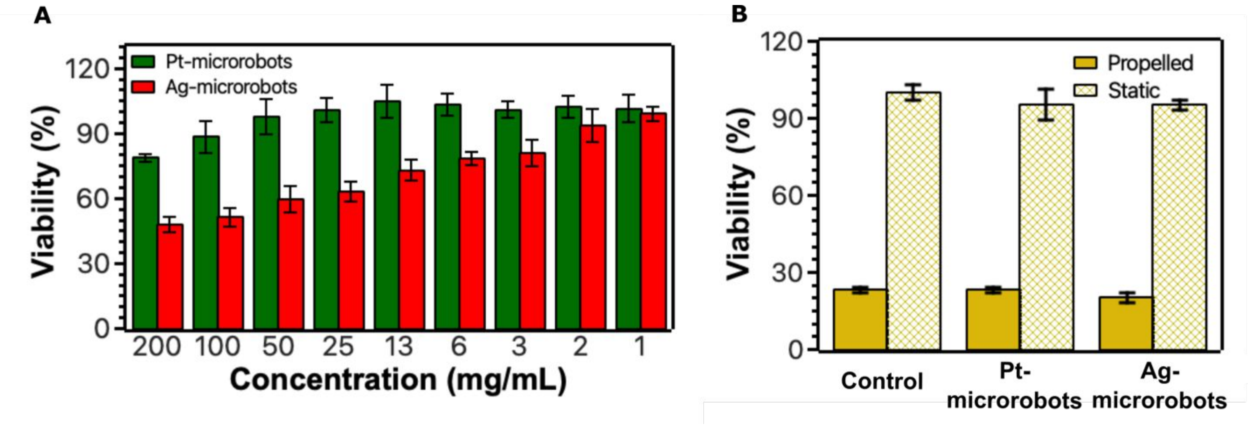

Figure S7. Viability of HaCaT cell culture in the presence of Pt-/Ag-microrobots. A) Viability of HaCaT cell culture as a function of the concentration of microrobots. The exposure was done for 72 hours without applying any fuel. B) Viability of HaCaT cell culture in the presence of propelled and static microrobots. The propulsion was achieved by an addition of 0.2 wt% H_2O_2 and UV irradiation. The exposure was done for 30 minutes.
